# Itch as a critical factor in impaired health-related quality of life in patients with plaque psoriasis achieving clear or almost-clear skin: Analysis of the single-arm, open-label, multicenter, prospective ProLOGUE study

**DOI:** 10.1016/j.jdin.2022.06.013

**Published:** 2022-06-22

**Authors:** Takuya Miyagi, Yasumasa Kanai, Kenta Murotani, Yukari Okubo, Masaru Honma, Satomi Kobayashi, Mariko Seishima, Yoko Mizutani, Hiroki Kitabayashi, Shinichi Imafuku

**Affiliations:** a Department of Dermatology, University of the Ryukyus, Okinawa, Japan; b Medical Affairs, Kyowa Kirin Co, Ltd, Tokyo, Japan; c Biostatistics Center, Kurume University, Fukuoka, Japan; d Department of Dermatology, Tokyo Medical University, Tokyo, Japan; e Department of Dermatology, Asahikawa Medical University, Hokkaido, Japan; f Department of Dermatology, Seibo International Catholic Hospital, Tokyo, Japan; g Department of Dermatology, Gifu University Graduate School of Medicine, Gifu, Japan; h Department of Dermatology, Fukuoka University Faculty of Medicine, Fukuoka, Japan

**Keywords:** absolute PASI, brodalumab, health-related quality of life, itching, plaque psoriasis, real-life patients, DLQI, Dermatology Life Quality Index, EQ-5D-5L UI, European Quality of Life 5-Dimension 5-Level Utility Index, HRQoL, health-related quality of life, NRS, Numerical Rating Scale, PASI, Psoriasis Area and Severity Index, PRO, patient-reported outcome, RCT, randomized controlled trial

## Abstract

**Background:**

Patients with psoriasis report impaired health-related quality of life (HRQoL; Dermatology Life Quality Index score ≥ 2) even after achieving clear or almost-clear skin with biologic treatment.

**Objective:**

To assess the effectiveness of brodalumab in HRQoL improvement and the factors associated with incomplete HRQoL improvement in Japanese patients with psoriasis.

**Methods:**

As a part of the single-arm, open-label, multicenter, prospective ProLOGUE study (Japan Registry of Clinical Trials identifier: jRCTs031180037), patients were treated with 210 mg of subcutaneous brodalumab in daily clinical practice until week 48. The absolute Psoriasis Area and Severity Index scores and patient-reported outcomes were assessed at baseline and weeks 12 and 48.

**Results:**

Seventy-three patients (male, 82.2%; median age, 54.0 years) were enrolled. The Dermatology Life Quality Index and European Quality of Life 5-Dimension 5-Level Utility Index scores significantly improved from baseline to weeks 12 and 48. At week 48, all 13 patients with a Dermatology Life Quality Index score of ≥2 and an absolute Psoriasis Area and Severity Index score of 0 to ≤2 reported itching.

**Limitations:**

Unclear generalizability of the results to other biologics.

**Conclusion:**

Treatment with brodalumab improved HRQoL in patients with psoriasis. Itching may contribute to incomplete HRQoL improvement in patients who have achieved clear or almost-clear skin.

**Capsule Summary**

• Treatment with brodalumab improved health-related quality of life (HRQoL) in Japanese patients with psoriasis.
• We recommend that patients with psoriasis who have achieved clear or almost-clear skin be monitored for the persistence of itching because itching may contribute to incomplete HRQoL improvement in these patients.

## Introduction

Psoriasis affects approximately 100 million people worldwide.^1^ Plaque psoriasis is the most common subtype of the disease and is characterized by itching, scaling, and pain,^2^ and an increase in the severity of these symptoms negatively affects the quality of life of patients.^3^

Previous randomized controlled trials (RCTs) have led to the approval of several biologic agents for the treatment of psoriasis.^4^^,^^5^ Findings from RCTs have demonstrated the efficacy of biologic agents in not only achieving clear or almost-clear skin but also improving health-related quality of life (HRQoL) in patients with psoriasis.^6^, ^7^, ^8^, ^9^, ^10^ Following biologic treatment, the proportion of patients who achieved a Dermatology Life Quality Index (DLQI) score of 0 and 1 (no effect on patients’ life)^11^ was consistently higher among patients with a higher Psoriasis Area and Severity Index (PASI) response (PASI score ≥90) than among patients with a lower PASI response.^6^, ^7^, ^8^, ^9^, ^10^ Nevertheless, a DLQI score of 0 and 1 could not be achieved in approximately 10% to 30% of patients who achieved a PASI score of ≥90,^6-10^ and the underlying causes of incomplete HRQoL improvement (DLQI score ≥2) in these patients have not been fully elucidated.

For the assessment of treatment response in patients with plaque psoriasis, absolute PASI is recommended as an alternative to PASI achievement rate (or relative PASI improvement) in view of the lack of absolute PASI scores at baseline.^12^^,^^13^ Approximately 30% to 40% of patients with psoriasis have an absolute PASI score of ≤10 and do not meet the eligibility criteria for RCTs in patients with moderate-to-severe plaque psoriasis,^14^^,^^15^ indicating the need for patient assessment using absolute PASI in real-life settings. An absolute PASI score of ≤2 or a PASI score of 90 is used as a suitable treatment target for psoriasis^16^, ^17^, ^18^; however, there is only limited research that suggests strategies for the improvement of HRQoL in patients who achieve the absolute PASI-based treatment target without complete HRQoL improvement.

Brodalumab, a human anti-interleukin 17 receptor monoclonal antibody, has shown efficacy in RCTs by reducing the severity of psoriasis and improving HRQoL.^19^^,^^20^ To assess its effectiveness in Japanese patients with plaque psoriasis, we conducted impacts on patient-reported outcomes by brodalumab for patients suffering from plaque psoriasis in clinical practice (ProLOGUE), a single-arm, open-label, multicenter, prospective study (Japan Registry of Clinical Trials identifier: jRCTs031180037).^15^^,^^18^ In the current analysis of the ProLOGUE study, we assessed the real-life effectiveness of brodalumab in the improvement of HRQoL (DLQI score and European Quality of Life 5-Dimension 5-Level [EQ-5D-5L]^21^ Utility Index [UI] score). We then investigated the factors associated with incomplete HRQoL improvement despite the achievement of clear or almost-clear skin in patients with psoriasis in terms of patient-reported outcomes (PROs).

## Methods

### Study design and patients

The design and key eligibility criteria for the ProLOGUE study have been described previously.^15^ This study was conducted from October 2017 to March 2020 at 15 facilities across Japan. Patients (age ≥18 years) who had plaque psoriasis without peripheral arthritis symptoms and who were judged by physicians as being eligible for the self-administration of brodalumab were included. This study was conducted in accordance with the Declaration of Helsinki and was reviewed and approved by the research ethics committee of each participating facility (approval number for the representative facility [Fukuoka University]: 2017M093; approval date: November 2, 2017). Following the enforcement of the Japanese act for the conduct of clinical research funded by pharmaceutical companies in April 2018, this study was reviewed and approved by the Certified Review Board of the Nippon Medical School Foundation (approval number: nms2018-0601-01; approval date: October 3, 2018). All patients provided written informed consent for participation in the study.^15^

### Interventions and measurements

The patients received 210 mg of subcutaneous brodalumab in daily clinical practice (day 1, at weeks 1 and 2, and once every 2 weeks thereafter) until week 48. No criteria were set for concomitant or prohibited therapies.^18^

The PRO scores, including the DLQI score, EQ-5D-5L UI score, Itch Numerical Rating Scale (NRS) score, Skin Pain NRS score, Generalized Anxiety Disorder-7 score,^22^ and Patient Health Questionnaire-8 score,^23^ were collected using an electronic PRO system before a medical examination at baseline and weeks 12 and 48. The levels of itching and skin pain in the previous 24 hours were assessed using the Itch NRS and Skin Pain NRS scores, respectively, with the scores ranging from 0 (no itch or pain) to 10 (worst imaginable itch or pain). The Generalized Anxiety Disorder-7 and Patient Health Questionnaire-8 scores were used to assess patient anxiety symptoms (total score ranging from 0 to 21) and depressive symptoms (total score ranging from 0 to 24), respectively, in the previous 2 weeks (higher scores indicated greater severity of symptoms). The Japanese tariff was used to calculate the EQ-5D-5L UI score and its dimension scores.^24^

### Outcomes

The primary outcomes of this analysis were changes in the DLQI and EQ-5D-5L UI scores from baseline to weeks 12 and 48. The exploratory outcomes included changes in the DLQI subscale scores and EQ-5D-5L dimension scores from baseline to weeks 12 and 48, DLQI score at weeks 12 and 48 in subgroups stratified by severity (absolute PASI 0 [clear skin], >0 to ≤2 [almost-clear skin], and >2), and characteristics of patients reporting incomplete HRQoL improvement at weeks 12 and 48. The DLQI scores were categorized into 3 groups (0, 1, and ≥2) based on their clinical interpretation (0-1, 2-5, 6-10, 11-20, and 21-30 for no, small, moderate, very large, and extremely large effect on the patient’s life, respectively).^11^

### Statistical analyses

All patients, except those who did not receive brodalumab and those with no evaluable efficacy data, were included in the analysis.

The DLQI score and its subscale scores, as well as the EQ-5D-5L UI score and its dimension scores, were compared between baseline and weeks 12 and 48 using the Wilcoxon’s signed rank test. The distribution of DLQI scores (0, 1, or ≥2) was summarized in 3 subgroups stratified by absolute PASI scores at weeks 12 and 48 (0, >0 to ≤2, and >2). The proportions of patients with a DLQI score of 0 and 1 and an absolute PASI score of ≤2 and patients with a DLQI score of 0 and 1 and an absolute PASI score of >2 were compared using the Fisher’s exact test. Patients with a DLQI score of ≥2 at weeks 12 and 48 were categorized into subgroups with an absolute PASI score of 0 and an absolute PASI score of >0 to ≤2, and the number and percentage of patients with psoriatic symptoms and lesion involvement were calculated for each subgroup.

Analyses were performed using last observation carried forward; discontinuations up to week 12 were recorded as week 12 data, and discontinuations after week 12 were recorded as week 48 data. No imputation was performed for other missing data. Statistical significance was defined as a 2-tailed *P* value of <.05. No statistical hypothesis was set, and multiplicity was not accounted for because all analyses were performed in an exploratory manner. All analyses were performed using SAS, version 9.4 (SAS Institute Inc.).

## Results

### Patient characteristics

The baseline characteristics of the 73 patients included in the ProLOGUE study have been reported previously.^15^ Briefly, 60 (82.2%) patients were men, and their median (quartile 1-quartile 3) age was 54 (44-64) years. Fingernail, toenail, and head involvement was observed in 46 (63.0%), 31 (42.5%), and 66 (90.4%) patients, respectively.^15^

Among the 73 patients at baseline, 56 completed the 48-week treatment. One patient withdrew from the study between baseline and week 12 because of lack of efficacy, and another 16 patients withdrew between weeks 12 and 48. At baseline and at weeks 12 and 48, a total of 73, 73, and 69 patients had evaluable PASI data and 73, 72, and 65 patients had complete PRO data, respectively.^18^

Thirty-four patients (46.6%) had comorbidities at baseline, with the major (incidence ≥ 5%) ones being hypertension (27.4%), hyperuricemia or gout (17.8%), diabetes mellitus (13.7%), dyslipidemia (13.7%), and hepatic steatosis or abnormal hepatic function (9.6%).^15^ Analyses of the DLQI scores between patients with major comorbidities and those without major comorbidities at baseline revealed no significant difference at baseline and at weeks 12 and 48 (Wilcoxon’s rank-sum test; data not shown). The concomitant therapies prescribed at least once during the study period included cyclosporine (3 patients), phototherapy (2 patients), methotrexate (1 patient), apremilast (1 patient), and etretinate (1 patient). Two patients used other nonbiologic agents and 58 used topical agents.

### Changes in DLQI and EQ-5D-5L UI scores from baseline

**Fig 1:**
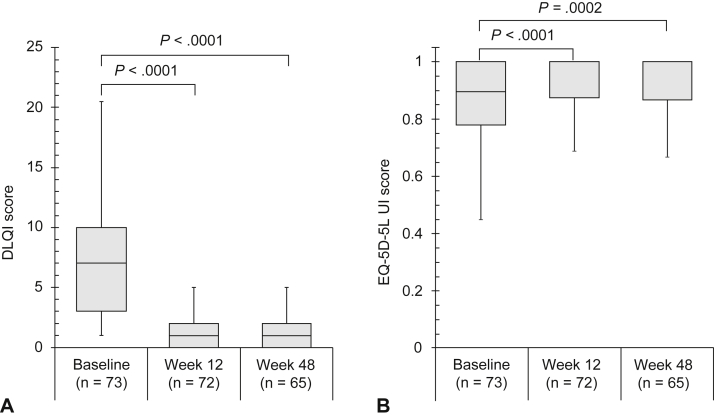
Plaque psoriasis. **(A)** Dermatology Life Quality Index and **(B)** European Quality of Life 5-Dimension 5 Level Utility Index scores at baseline and at weeks 12 and 48. Tested using the Wilcoxon’s signed rank test. The top whisker represents quartile (Q)3 + (1.5 × interquartile range) or the maximum, whichever is lower. The bottom whisker represents Q1 − (1.5 × interquartile range) or the minimum, whichever is higher. The top border of the box represents Q3; the bottom border represents Q1; and the middle line represents the median. *DLQI*, Dermatology Life Quality Index; *EQ-5D-5L UI*, European Quality of Life 5-Dimension 5-Level Utility Index.

The DLQI score significantly decreased from baseline to weeks 12 and 48 (*P* < .0001 for both) of brodalumab treatment (Fig 1, *A*). The EQ-5D-5L UI score significantly increased from baseline to weeks 12 (*P* < .0001) and 48 (*P* = .0002) of brodalumab treatment (Fig 1, *B*). All DLQI subscale scores significantly changed from baseline to weeks 12 and 48, as did the EQ-5D-5L dimension scores of usual activity, pain or discomfort, and anxiety or depression (Table I).

**Table I:** Dermatology Life Quality Index subscale scores and European Quality of Life 5-Dimension 5 Level dimension scores at baseline and weeks 12 and 48∗

Variable	Baseline (n = 73)	Wk 12 (n = 72)	*P* value	Wk 48 (n = 65)	*P* value
DLQI subscale scores					
Symptoms and feelings	3.0 (2.0-4.0)	1.0 (0.0-1.0)	<.0001	1.0 (0.0-1.0)	<.0001
Daily activities	1.0 (0.0-2.0)	0.0 (0.0-0.5)	<.0001	0.0 (0.0-0.0)	<.0001
Leisure	1.0 (0.0-2.0)	0.0 (0.0-0.0)	<.0001	0.0 (0.0-0.0)	<.0001
Work and school	0.0 (0.0-1.0)	0.0 (0.0-0.0)	.0003	0.0 (0.0-0.0)	.0335
Personal relationships	0.0 (0.0-0.0)	0.0 (0.0-0.0)	.0016	0.0 (0.0-0.0)	.0162
Treatment	1.0 (0.0-2.0)	0.0 (0.0-0.0)	<.0001	0.0 (0.0-0.0)	<.0001
EQ-5D-5L dimension scores					
Mobility	1.0 (1.0-1.0)	1.0 (1.0-1.0)	1.0000	1.0 (1.0-1.0)	.6790
Self-care	1.0 (1.0-1.0)	1.0 (1.0-1.0)	.7969	1.0 (1.0-1.0)	.6719
Usual activity	1.0 (1.0-2.0)	1.0 (1.0-1.0)	.0144	1.0 (1.0-1.0)	.0049
Pain or discomfort	2.0 (1.0-2.0)	1.0 (1.0-2.0)	<.0001	1.0 (1.0-2.0)	<.0001
Anxiety or depression	1.0 (1.0-2.0)	1.0 (1.0-1.0)	.0179	1.0 (1.0-1.0)	.0019

*DLQI,* Dermatology Life Quality Index; *EQ-5D-5L,* European Quality of Life 5-Dimension 5-Level.

∗ The data are presented as median (quartile 1-quartile 3), tested using the Wilcoxon’s signed rank test.

### DLQI score in subgroups stratified by absolute PASI

**Fig 2:**
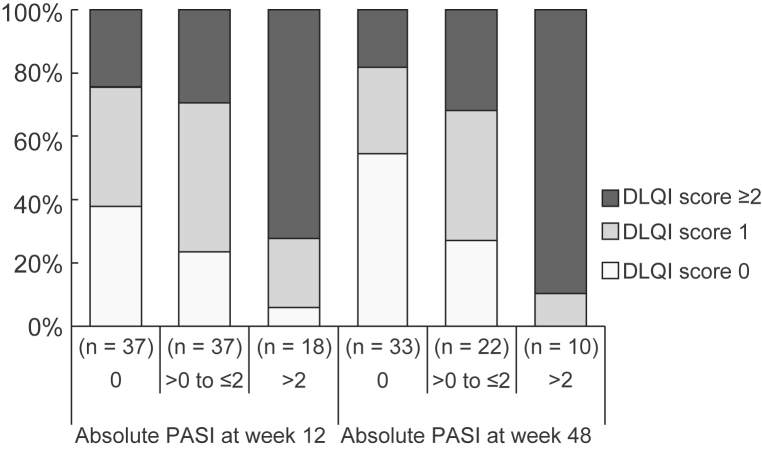
Plaque psoriasis. Distribution of Dermatology Life Quality Index scores at weeks 12 and 48 stratified by severity. *DLQI*, Dermatology Life Quality Index; *PASI*, Psoriasis Area and Severity Index.

At week 12, 37.5% (27/72) of the patients had a DLQI score of ≥2. Notably, 24.3% (9/37) of patients with an absolute PASI score of 0 at week 12 had a DLQI score of ≥2, whereas 27.8% (5/18) of patients with an absolute PASI score of >2 reported a DLQI score of 0 and 1 (Fig 2). Similarly, at week 48, 33.8% (22/65) of the patients had a DLQI score of ≥2, and 18.2% (6/33) of patients with an absolute PASI score of 0 had a DLQI score of ≥2 (Fig 2). The proportion of patients with a DLQI score of 0 and 1 and an absolute PASI score of ≤2 was significantly higher than that of patients with a DLQI score of 0 and 1 and an absolute PASI score of >2, at both week 12 (55.6% [40/72 patients] vs 6.9% [5/72 patients]; *P* = .0007) and week 48 (64.6% [42/65 patients] vs 1.5% [1/65 patients]; *P* = .0001).

### Characteristics of patients reporting incomplete HRQoL improvement following brodalumab treatment

Fourteen and 13 patients with a DLQI score of ≥2 and an absolute PASI score of 0 to ≤2 at weeks 12 and 48, respectively, were assessed for the presence of symptoms and lesion involvement. Six of the 13 patients with a DLQI score of ≥2 at week 48 had a DLQI score of ≥2 at week 12 as well. The most commonly (>50%) observed symptoms and lesion involvements at week 12 were itching (88.9%) in patients with an absolute PASI score of 0 and fingernail and head involvement (both 80.0%) in patients with an absolute PASI score of >0 to ≤2; the most commonly (>50%) observed symptoms and lesion involvement at week 48 were itching (100.0%) in patients with an absolute PASI score of 0 and itching (100.0%), skin pain (71.4%), and head involvement (71.4%) in patients with an absolute PASI score of >0 to ≤2 (Table II).

**Table II:** Characteristics of patients with a Dermatology Life Quality Index score of ≥2 at weeks 12 and 48∗

Variable	Absolute PASI at wk 12		Absolute PASI at wk 48	
	0 (n = 9)	>0 to ≤2 (n = 5)	0 (n = 6)	>0 to ≤2 (n = 7)
Symptoms				
Itching†	8 (88.9)	2 (40.0)	6 (100.0)	7 (100.0)
Skin pain‡	4 (44.4)	1 (20.0)	3 (50.0)	5 (71.4)
Anxiety symptoms§	1 (11.1)	2 (40.0)	1 (16.7)	2 (28.6)
Depressive symptomsװ	2 (22.2)	3 (60.0)	1 (16.7)	2 (28.6)
Peripheral arthritis	0 (0.0)	2 (40.0)	1 (16.7)	0 (0.0)
Lesion involvement				
Fingernail	3 (33.3)	4 (80.0)	2 (33.3)	2 (28.6)
Toenail	2 (22.2)	2 (40.0)	0 (0.0)	1 (14.3)
Head	0 (0.0)	4 (80.0)	0 (0.0)	5 (71.4)
Intergluteal cleft	0 (0.0)	1 (20.0)	0 (0.0)	0 (0.0)
Lower portion of the leg	0 (0.0)	3 (60.0)	0 (0.0)	2 (28.6)
Back of the hand	0 (0.0)	1 (20.0)	0 (0.0)	2 (28.6)
Forearm	0 (0.0)	0 (0.0)	0 (0.0)	1 (14.3)

*PASI*, Psoriasis Area and Severity Index.

∗ The data are presented as n (%).

† Itch Numerical Rating Scale score ≥ 1.

‡ Skin pain Numerical Rating Scale score ≥ 1.

§ Generalized Anxiety Disorder-7 score ≥ 5.

װ Patient Health Questionnaire-8 score ≥ 5.

## Discussion

To identify the causes of incomplete HRQoL improvement after the achievement of clear or almost-clear skin in patients with psoriasis, we assessed the effectiveness of brodalumab in improving patients’ HRQoL and the factors leading to incomplete HRQoL improvement using PRO instruments.

In this analysis, a majority of the patients reported a DLQI score of 0 and 1 (no effect on patient’s life^11^) after brodalumab treatment (62.5% at week 12; 66.2% at week 48), and some reported a DLQI score of 0 and 1 without achieving an absolute PASI score of ≤2. In contrast, 24.3% and 18.2% of patients who achieved an absolute PASI score of 0 reported a DLQI score of ≥2 at weeks 12 and 48, respectively. An absolute PASI score of ≤2 has been reported to be a clinically significant treatment goal in terms of both clear skin and HRQoL.^12^^,^^16^ Our results reiterate the importance of a DLQI score of 0 and 1 as one of the treatment goals in daily clinical practice, independent of PASI.

The clinical characteristics of patients with incomplete HRQoL improvement despite biologic treatment have not been fully elucidated. Notably, itching was reported in a majority of patients with a DLQI score of ≥2 and an absolute PASI score of 0 (88.9% at week 12; 100.0% at week 48) or >0 to ≤2 (100.0% at week 48) in the current analysis, suggesting the involvement of itching in incomplete HRQoL improvement in patients who achieved clear or almost-clear skin. Moreover, a majority of patients with a DLQI score of ≥2 and an absolute PASI score of >0 to ≤2 had residual psoriatic lesions on the head (80.0% at week 12; 71.4% at week 48) or fingernails (80.0% at week 12), which indicates the burden of head and nail psoriasis on the HRQoL of patients with almost-clear skin. Indeed, nail involvement in psoriasis is quite common (48.1%),^25^ and the prevalence of fingernail psoriasis in patients who achieved clear or almost-clear skin with a DLQI score of ≥2 in the current study (28.6%-80.0%) was higher than that reported previously in patients with clear skin (1%-5%).^26^ Because psoriasis of the head and nails is among the most difficult-to-control lesions visible to others and significantly impairs the quality of life of affected patients,^27^, ^28^, ^29^ monitoring residual psoriatic lesions of the head and nails may be important in patients who achieve almost-clear skin.

Itching in patients with psoriasis has not been well studied because psoriasis has historically been considered a nonpruritic disease, unlike atopic dermatitis.^30^ However, itching is one of the most burdensome symptoms associated with psoriasis^31^ and the most important factor contributing to the severity of psoriasis.^32^ Previous RCT-based findings of biologics in patients with psoriasis have indicated that improvement in itching, as well as PASI response, plays an important role in improving patient HRQoL,^33^^,^^34^ which is consistent with our findings in the real-life setting. The involvement of multiple mediators and receptors in itching in patients with psoriasis has been suggested, such as transient receptor potential melastatin 8, transient receptor potential vanilloid 3,^35^ and keratinocyte-related signals (eg, protein gene product 9.5 and keratin 16).^36^^,^^37^ Therefore, in patients with psoriasis, signals relevant to itching may not be fully eliminated by biologic treatment even after clear skin is achieved. Because a DLQI score of 0 and 1, independent of PASI, could be a treatment goal in daily clinical practice from a patient perspective, patients who have achieved clear or almost-clear skin should be monitored for the persistence of itching for complete DLQI improvement, particularly in long-term treatment.

Our study has some limitations. First, analyses stratified by concomitant medication or other factors were not feasible because of the small sample size. Second, because the study focused on intervention with brodalumab, the generalizability of the results to other biologic agents may be limited. Third, sex-based differences were not assessed because of the limited number of female patients enrolled. The impact of sex warrants further investigation because DLQI and its subscale scores among patients with psoriasis tend to be more seriously impaired in women than in men.^38^ Lastly, although HRQoL is often impaired in patients with psoriasis by non–skin-primary comorbidities (eg, anxiety disorder, hypertension, and obesity),^39^ our analyses did not consider the adjustment of these factors.

## Conclusion

Treatment with brodalumab improved HRQoL in Japanese patients with psoriasis in a real-life setting. Our results suggest that itching may contribute to incomplete HRQoL improvement in patients who achieve clear or almost-clear skin following brodalumab treatment.

## Conflicts of interest

Dr Miyagi reports financial support from Kyowa Kirin during the conduct of the study; is a consultant or advisor for AbbVie and Janssen Pharmaceuticals; has received funding or grants from AbbVie, Eisai, and Taiho Pharmaceutical; has received speaking and lecture fees from AbbVie, Janssen Pharmaceuticals, Maruho, Novartis Pharma, Boehringer Ingelheim, Eisai, Eli Lily Japan, CSL Behring, Taiho Pharmaceutical, Ono Pharmaceutical, Otsuka Pharmaceutical, and LEO Pharma; and has received travel reimbursement from AbbVie, Janssen Pharmaceuticals, Maruho, Novartis Pharma, Taiho Pharmaceutical, and Ono Pharmaceutical outside the submitted work. Dr Kanai is an employee of Kyowa Kirin. Dr Murotani reports financial support from Kyowa Kirin during the conduct of the study. Dr Okubo reports financial support from Kyowa Kirin during the conduct of the study; is a consultant or advisor for AbbVie, Amgen, Boehringer Ingelheim, Kyowa Kirin, Sun Pharma, UCB Pharma, Taiho Pharmaceutical, Eli Lilly Japan, Novartis Pharma, Maruho, LEO Pharma, and Janssen Pharmaceuticals; has received funding or grants from AbbVie, Eisai, Kaken Pharmaceutical, Kyowa Kirin, GlaxoSmithKline Shiseido, Sun Pharma, JIMRO, Taiho Pharmaceutical, Torii Pharmaceutical, Eli Lilly Japan, Novartis Pharma, Maruho, LEO Pharma, and Janssen Pharmaceuticals; and has received speaking and lecture fees from AbbVie, Amgen, Boehringer Ingelheim, Eisai, Kyowa Kirin, Sanofi, Sun Pharma, UCB Pharma, JIMRO, Taiho Pharmaceutical, Mitsubishi Tanabe Pharma, Torii Pharmaceutical, Eli Lilly Japan, Novartis Pharma, Maruho, LEO Pharma, Celgene, and Janssen Pharmaceuticals outside the submitted work. Dr Honna reports financial support from Kyowa Kirin during the conduct of the study; has received funding or grants from Kyowa Kirin and Eli Lilly Japan; and has received speaking and lecture fees from Kyowa Kirin, Novartis Pharma, Maruho, Eisai, Janssen Pharmaceuticals, Taiho Pharmaceutical, Mitsubishi Tanabe Pharma, AbbVie, Torii Pharmaceutical, Eli Lilly Japan, Sun Pharma, and Celgene outside the submitted work. Dr Kobayashi reports statistical analysis support and writing assistance from Kyowa Kirin during the conduct of the study and has received speaking and lecture fees from Janssen Pharmaceuticals and Taiho Pharmaceutical outside the submitted work. Dr Seishima reports financial support from Kyowa Kirin during the conduct of the study; has received funding or grants from Taiho Pharmaceutical, Novartis Pharma, Sanofi, Maruho, Mitsubishi Tanabe Pharma, Kyowa Kirin, Sun Pharma, Kaken Pharmaceutical, Japan Blood Products Organisation, Eisai, and Torii Pharmaceutical; has received speaking and lecture fees from Taiho Pharmaceutical, Novartis Pharma, Sanofi, Maruho, Eli Lilly Japan, Mitsubishi Tanabe Pharma, Kyowa Kirin, Sun Pharma, Kaken Pharmaceutical, LEO Pharma, Celgene, Janssen Pharmaceuticals, Eisai, AbbVie, Torii Pharmaceutical, and JIMRO; and has received travel reimbursement from Taiho Pharmaceutical, Novartis Pharma, Sanofi, Maruho, Eli Lilly Japan, Mitsubishi Tanabe Pharma, Kyowa Kirin, Sun Pharma, Kaken Pharmaceutical, Japan Blood Products Organisation, LEO Pharma, Celgene, Janssen Pharmaceuticals, Pola Pharma, Eisai, AbbVie, Torii Pharmaceutical, and JIMRO outside the submitted work. Dr Mizutani reports financial support, article publishing charges, statistical analysis support, and writing assistance from Kyowa Kirin during the conduct of the study and has received speaking and lecture fees from Taiho Pharmaceutical, Novartis Pharma, Sanofi, Maruho, Eli Lilly Japan, Mitsubishi Tanabe Pharma, Sun Pharma, Kaken Pharmaceutical, Japan Blood Products Organisation, and Kyowa Kirin outside the submitted work. Author Kitabayashi is an employee of Kyowa Kirin and owns stock in the company. Dr Imafuku reports financial support from Kyowa Kirin during the conduct of the study; has received speaking and lecture fees from Astellas Pharma, AbbVie, Eli Lilly Japan, Otsuka Pharmaceutical, Kaken Pharmaceutical, Sato Pharmaceutical, Sanofi, Sun Pharma, GlaxoSmithKline, Celgene, Daiichi Sankyo, Takeda Pharmaceutical, Taiho Pharmaceutical, Mitsubishi Tanabe Pharma, Tsumura, Torii Pharmaceutical, Nippon Zoki Pharmaceutical, Novartis Pharma, Pfizer, Bristol Myers Squibb, Maruho LEO Pharma, Janssen Pharmaceuticals, and UCB Japan; has received funding or grants from AbbVie, Eisai, Sato Pharmaceutical, Sun Pharma, Taiho Pharmaceutical, Mitsubishi Tanabe Pharma, Tsumura, Maruho, and LEO Pharma; and is a consultant or advisor for Bristol, Meiji Seika Pharma, and LEO Pharma outside the submitted work.
